# Supplementary material for: Unraveling the phylogenomic diversity of Methanomassiliicoccales and implications for mitigating ruminant methane emissions
Source: Genome Biol. 2024 Jan 23;25:32. doi: 10.1186/s13059-024-03167-0 (PMC10804542; doi:10.1186/s13059-024-03167-0)
Supplement: Supplementary file 1 — Additional file 1: Supplementary Figures. This additional file contains the supplementary figures (Figs. S1-S6). [file 13059_2024_3167_MOESM1_ESM.docx]

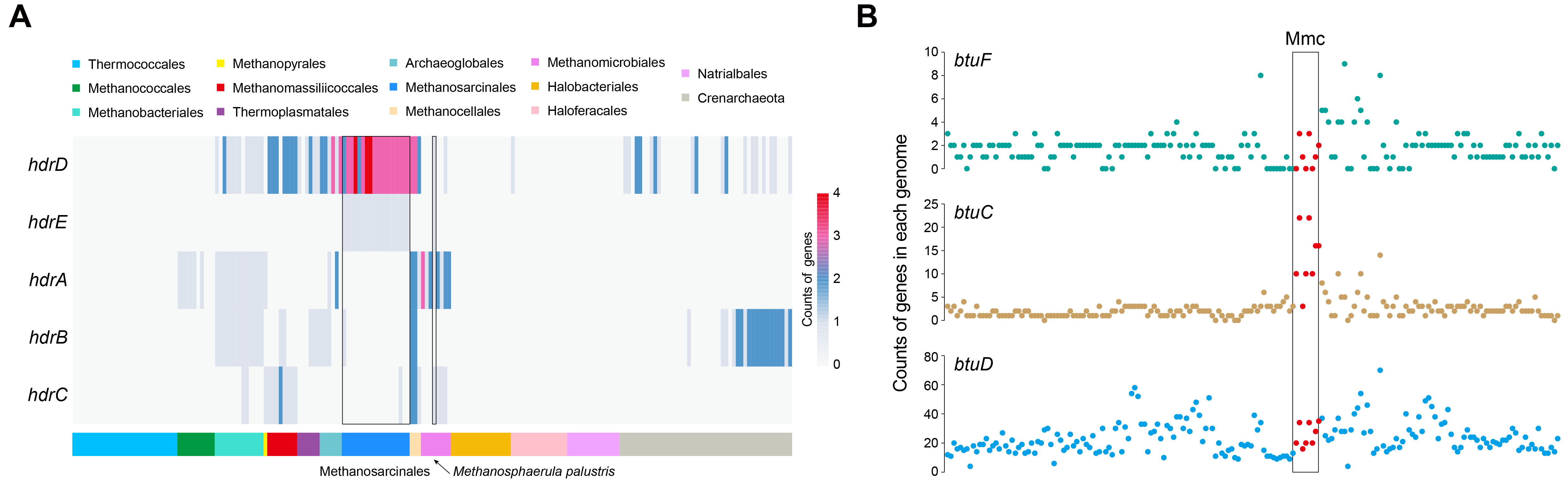


**Fig. S1. A** Heatmap generated using gene counts for *hdrD*, *hdrE*, *hdrA*, *hdrB*, and *hdrC* across all 192 genomes included in the analysis, with the colored bars corresponding to the respective genera to which each genome belongs. **B** Gene copy numbers of *btuF*, *btuC*, and *btuD* in all 192 included genomes. Red circles represent the genome of Methanomassiliicoccales. Mmc, Methanomassiliicoccales.


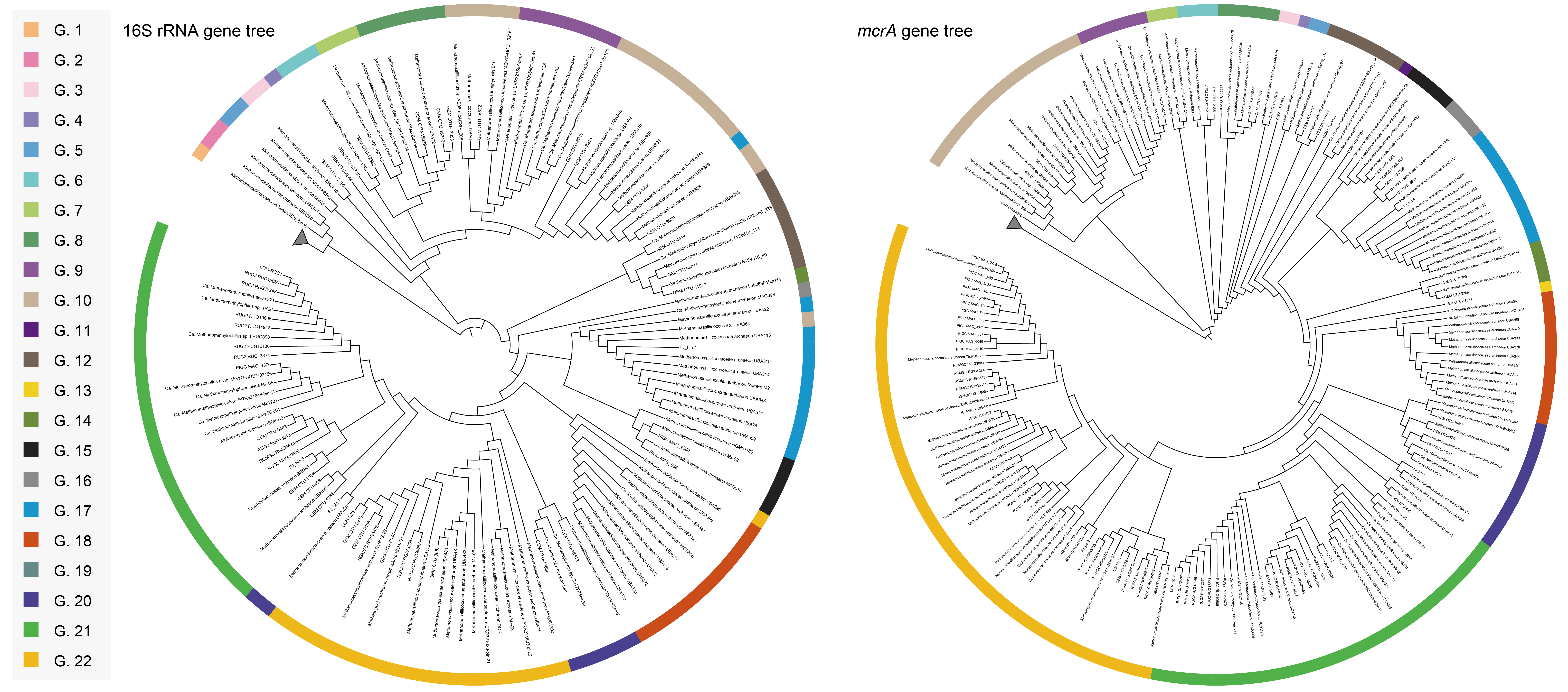


**Fig. S2.** Maximum likelihood trees based on the 16S rRNA and *mcrA* genes extracted from the Methanomassiliicoccales genomes was constructed to support the taxonomy. Colored strips show the genus to which the genome belongs. The gray triangle corresponds to the outgroup taxa.


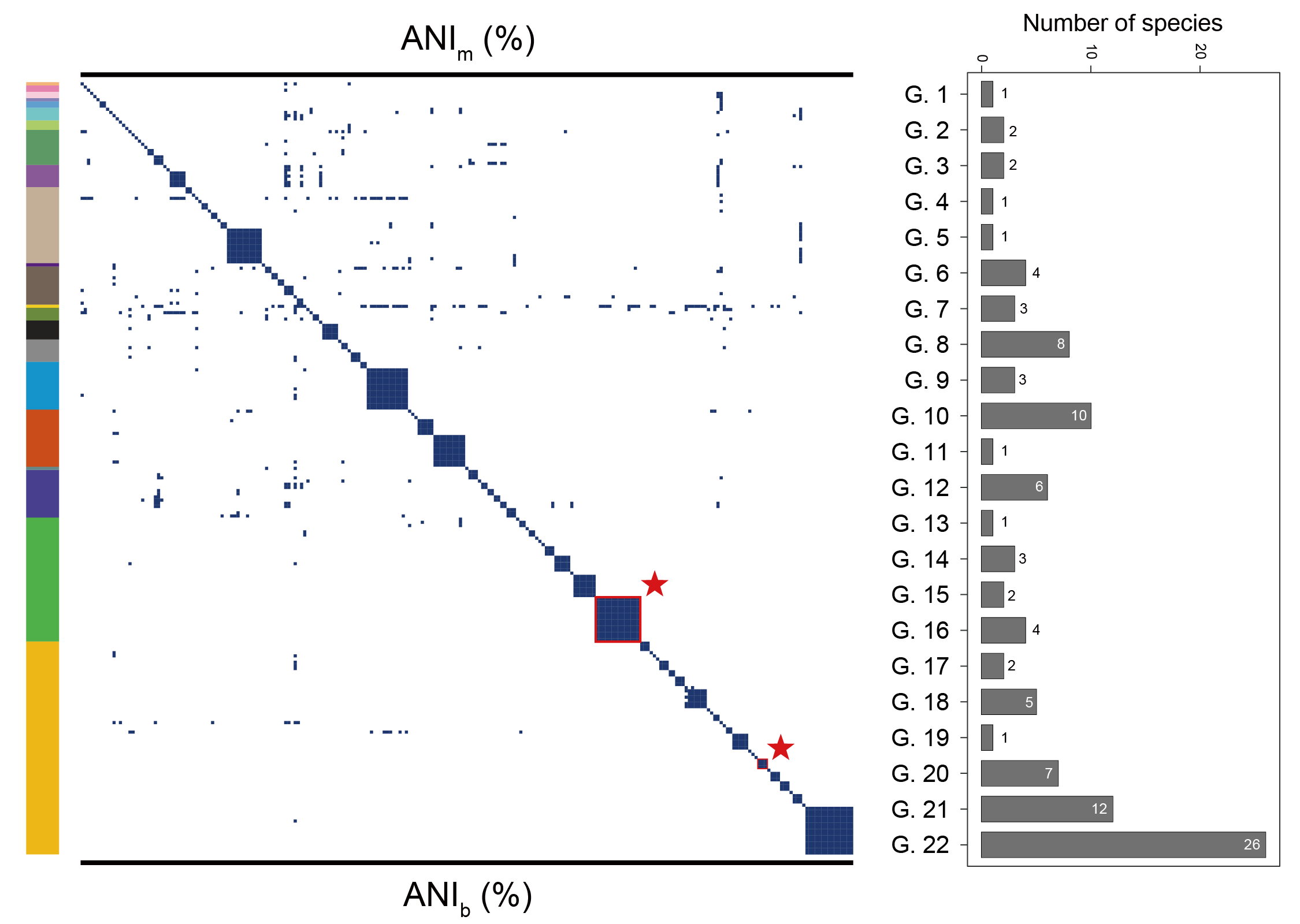


**Fig. S3.** The distribution of 105 species among different genera. The stars on the heatmap represent the two Methanomassiliicoccales strains in this study, LGM-RCC1 and LGM-DZ1. The ANIm and ANIb values >95% are shown.


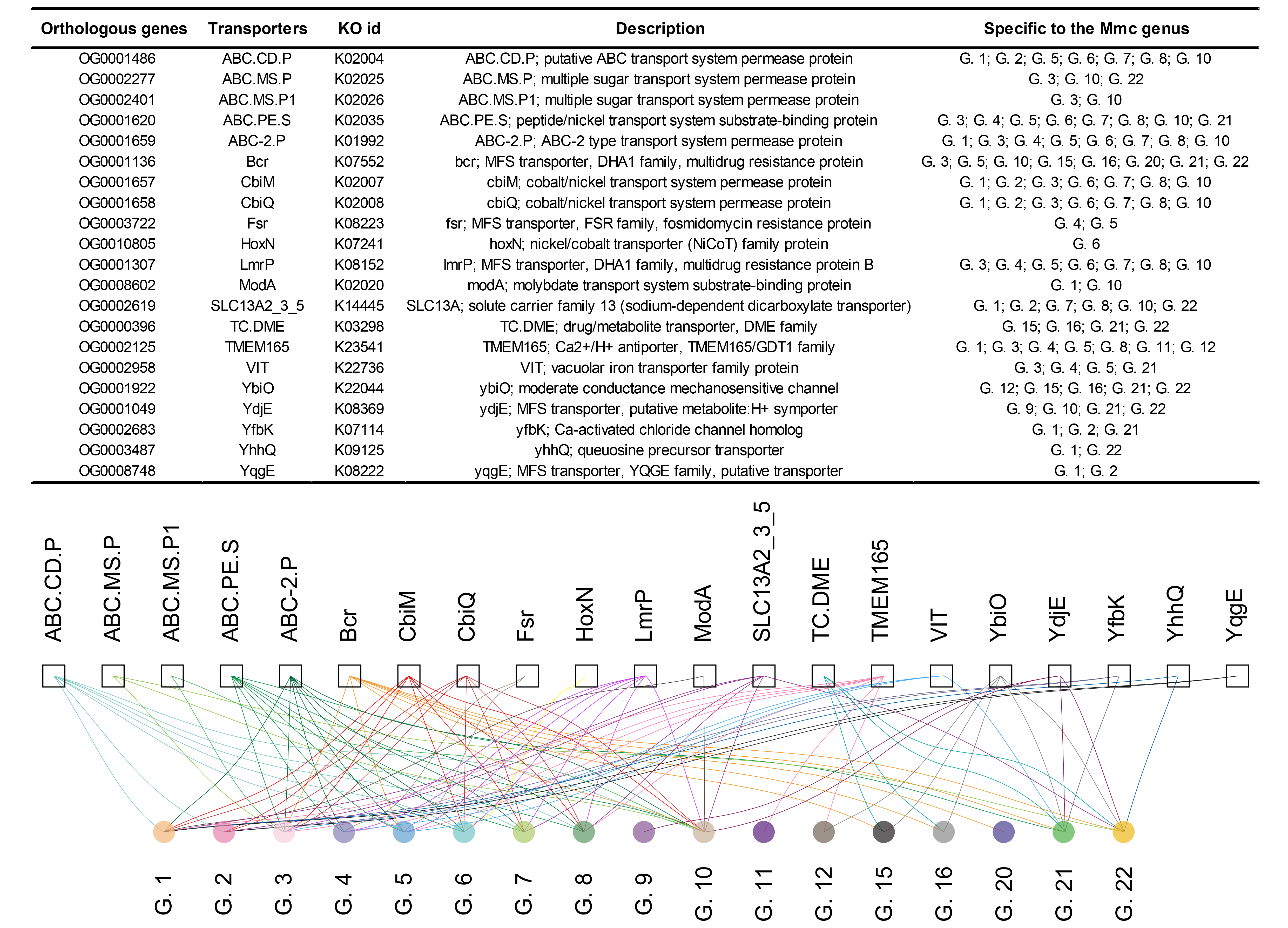


**Fig. S4.** Detailed gene information and genus distribution of 21 genes involved in transporters.


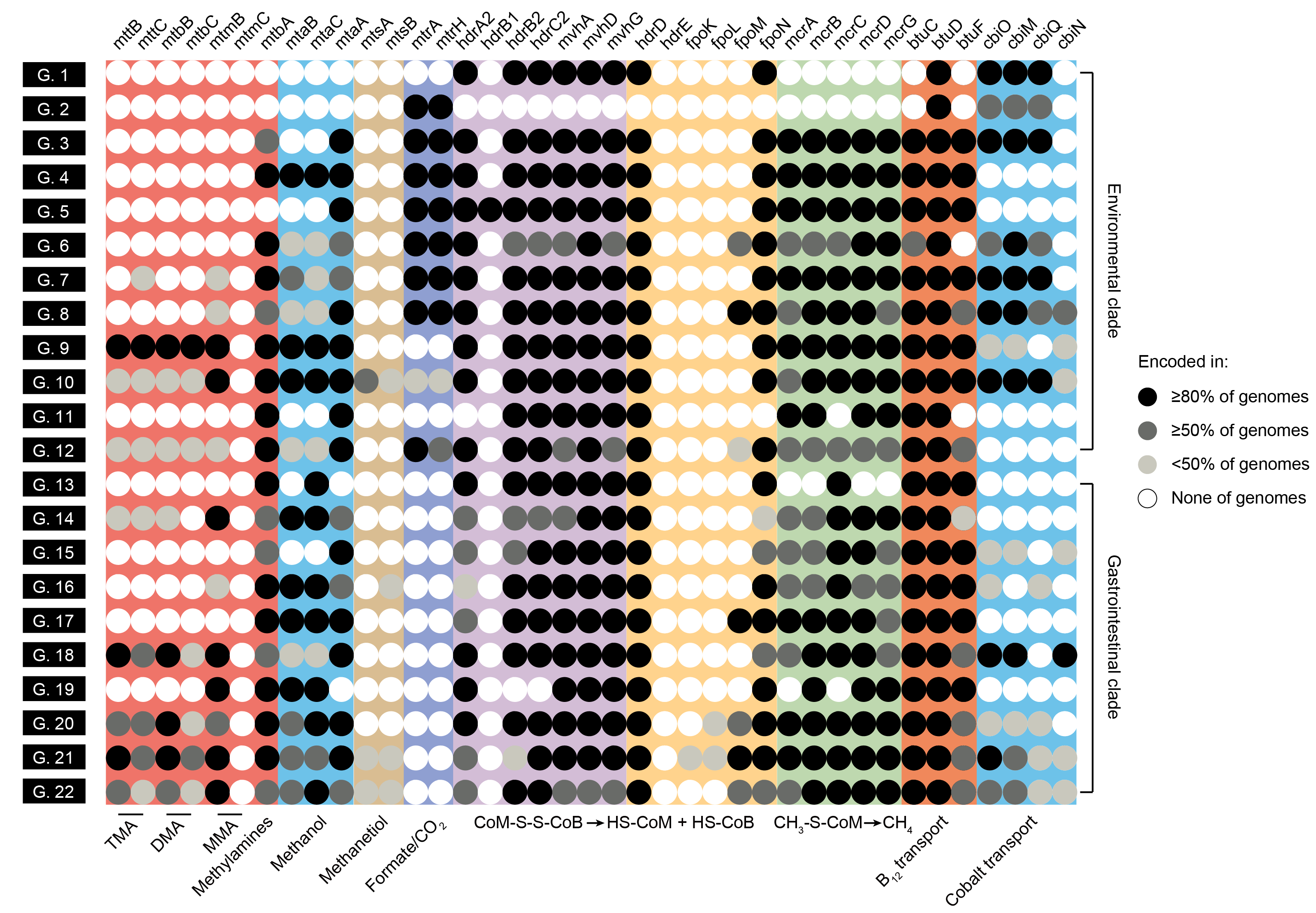


**Fig. S5.** Circle colors indicate the frequency of the genes present in all the genomes of the corresponding genus. The gene names for each column are shown at the top, and the processes they are involved in are shown at the bottom.


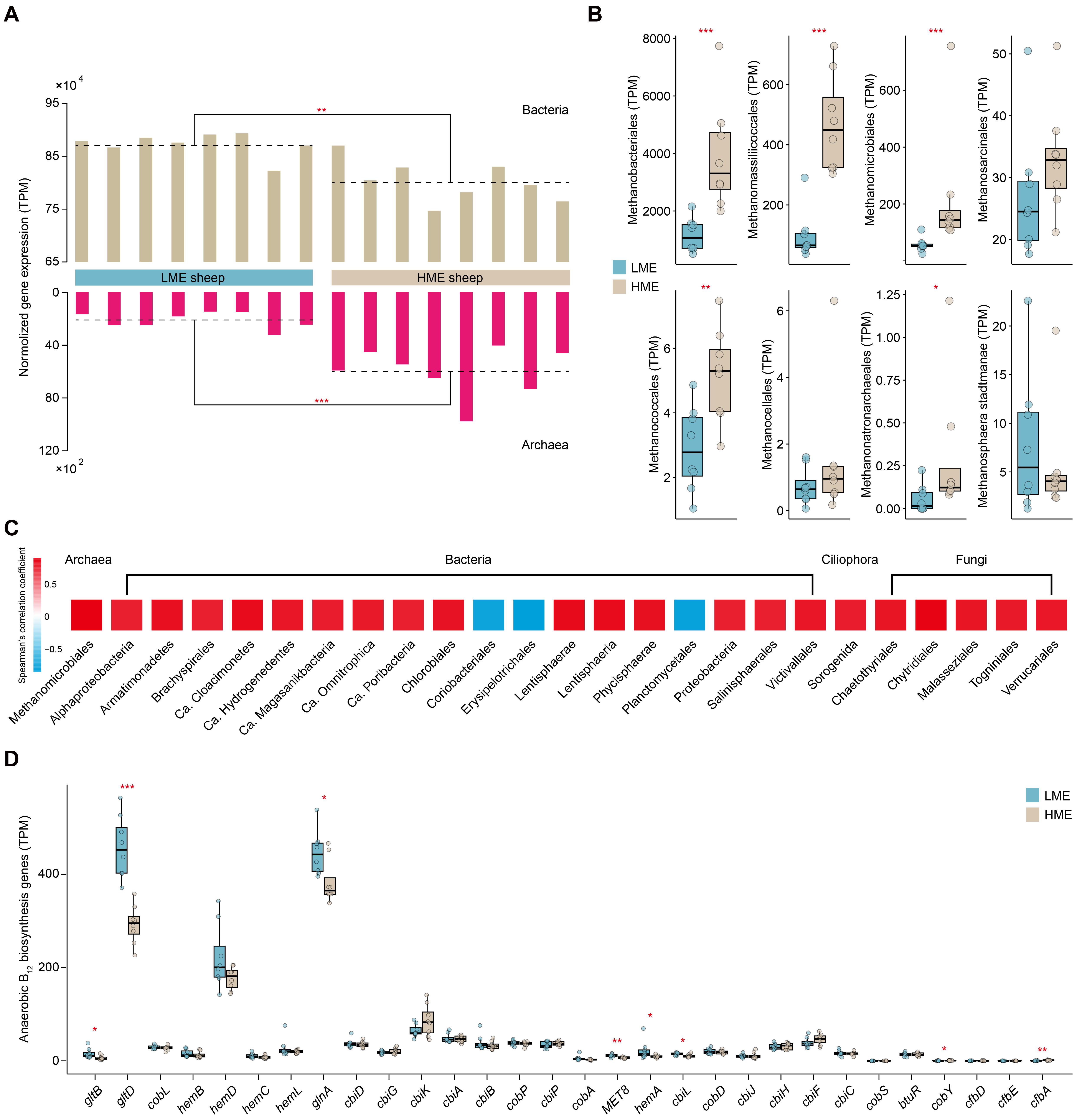


**Fig. S6.** **A** The bar plot shows the total gene expression of rumen bacterial and archaeal communities in the LME and HME sheep. **B** The differences in gene expression for various methanogenic orders, as well as *Methanosphaera stadtmanae*, between the LME and HME groups. **C** The heatmap displayed correlations between Methanomassiliicoccales and specific microorganisms at the order level, highlighting 25 orders with significant correlations. **D** The differences in gene expression for the anaerobic B_12_ biosynthesis pathway in the rumen microbiome between the LME and HME groups were examined. The significance level between the two groups has been compared. Wilcoxon rank-sum test, **P* < 0.05, ***P* < 0.01, ****P* < 0.001.
